# Brain‐based sex differences in schizophrenia: A systematic review of fMRI studies

**DOI:** 10.1002/hbm.26664

**Published:** 2024-03-23

**Authors:** Mohammad Amin Salehi, Rasa Zafari, Soheil Mohammadi, Mohammad Shahrabi Farahani, Mahsa Dolatshahi, Hamid Harandi, Amirhossein Poopak, Stephen R. Dager

**Affiliations:** ^1^ School of Medicine Tehran University of Medical Sciences Tehran Iran; ^2^ Medical Students Research Committee Shahed University Tehran Iran; ^3^ Mallinckrodt Institute of Radiology, Division of Neuroradiology Washington University in St. Louis St. Louis Missouri USA; ^4^ Department of Radiology University of Washington Seattle Washington USA

**Keywords:** functional MRI, neuroimaging, resting‐state fMRI, schizophrenia, sex difference, task‐based fMRI

## Abstract

Schizophrenia is a chronic psychiatric disorder with characteristic symptoms of delusions, hallucinations, lack of motivation, and paucity of thought. Recent evidence suggests that the symptoms of schizophrenia, negative symptoms in particular, vary widely between the sexes and that symptom onset is earlier in males. A better understanding of sex‐based differences in functional magnetic resonance imaging (fMRI) studies of schizophrenia may provide a key to understanding sex‐based symptom differences. This study aimed to summarize sex‐based functional magnetic resonance imaging (fMRI) differences in brain activity of patients with schizophrenia. We searched PubMed and Scopus to find fMRI studies that assessed sex‐based differences in the brain activity of patients with schizophrenia. We excluded studies that did not evaluate brain activity using fMRI, did not evaluate sex differences, and were nonhuman or in vitro studies. We found 12 studies that met the inclusion criteria for the current systematic review. Compared to females with schizophrenia, males with schizophrenia showed more blood oxygen level‐dependent (BOLD) activation in the cerebellum, the temporal gyrus, and the right precuneus cortex. Male patients also had greater occurrence of low‐frequency fluctuations in cerebral blood flow in frontal and parietal lobes and the insular cortex, while female patients had greater occurrence of low‐frequency fluctuations in the hippocampus, parahippocampus, and lentiform nucleus. The current study summarizes fMRI studies that evaluated sex‐based fMRI brain differences in schizophrenia that may help to shed light on the underlying pathophysiology and further understanding of sex‐based differences in the clinical presentation and course of the disorder.

## Graphical abstract

Males with schizophrenia showed more activation in the cerebellum, the temporal gyrus, and the right precuneus cortex. Considering low‐frequency fluctuations, males showed greater occurrence in frontal and parietal lobes and the insular cortex, compared to females showing a higher range of low‐frequency fluctuations in the hippocampus, parahippocampus, and lentiform nucleus.

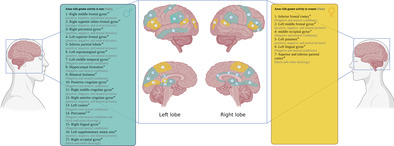

## 1 INTRODUCTION

Schizophrenia is a chronic psychiatric disorder with almost a 1% prevalence worldwide (Saha et al., 2005). The diagnosis is characterized by two main categories of symptoms: positive symptoms (including hallucinations, disordered thinking, and delusions) and negative symptoms (including self‐neglect, lack of motivation, paucity of thought, and social withdrawal) (Picchioni & Murray, 2007), while other symptom domains like cognitive dysfunction and psychomotor symptoms can contribute to a better assessment of schizophrenia (Blum et al., 2015; Bowie & Harvey, 2006; Morrens et al., 2008). Schizophrenia typically presents during later childhood or in early adulthood and has persistent symptoms in more than 50% of patients (Cooper & Michels, 1988), contributing to 13.4 million years of healthy life lost due to disability (Charlson et al., 2018). Approximately 20%–50% of patients with schizophrenia are resistant to treatment, with a resultant poorer prognosis, increasing the societal burden of this disorder (Lieberman, 1999).

Recent evidence suggests that the occurrence of schizophrenia varies substantially between the sexes (Gogos et al., 2015; Ochoa et al., 2012; Sun et al., 2016). The prevalence rate of schizophrenia in males is 1.4 times higher than for women (McGrath et al., 2004) and the presentation of symptoms is generally earlier for males (Galderisi et al., 2012; Häfner, 2003). There may be a protective effect of female sex hormones as the incidence of schizophrenia rises in women following menopause, in conjunction with decreasing estrogen and progesterone levels (Häfner, 2003; Sun et al., 2016). Interestingly, it has also been shown that negative symptoms, which are associated with a poorer prognosis, may be less frequent in premenopausal females, but increase during menopause (Galderisi et al., 2012; Morgan et al., 2008). Also, males are reported to be more resistant to treatment and more vulnerable to social problems, such as employment difficulties, which may worsen their prognosis (Cotton et al., 2009; Szymanski et al., 1995; Vila‐Rodriguez et al., 2011).

Sex‐based differences in brain morphometry and function have been reported in schizophrenia (Rubin et al., 2018). Increasingly, functional differences are being studied using fMRI. Functional magnetic resonance imaging (fMRI) is a neuroimaging method that allows semi‐quantitative detection of regional variations in blood oxygen level‐dependent (BOLD) signal as a measure of localized brain metabolic activity level (Raichle & Mintun, 2006). Task‐based fMRI is an fMRI approach that can evaluate regional brain metabolic changes during carefully controlled tasks, such as motor, visual, auditory, and cognitive tasks (Glover, 2011). A passive functional imaging approach, resting state fMRI, utilizes low‐frequency fluctuations of oxygenated and de‐oxygenated BOLD signal to assess regional patterns of synchronicity, or dys‐synchronicity, in metabolic activity in order to characterize brain networks (Smitha et al., 2017). Unlike task‐based fMRI, for resting state approaches there is no need to perform explicit tasks. Thus, research participants who may not be able to perform the tasks, such as infants and patients with low consciousness or agitation, can be evaluated during natural sleep (Fox & Greicius, 2010).

The aim of this study was to systematically review fMRI literature that specifically evaluated sex‐based brain activation differences in schizophrenia to provide a comprehensive overview of differences. Further, we assessed whether differences in sex‐based patterns of brain activation in might hold clues for better understanding schizophrenia more generally. Findings from this study additionally contribute to a better appreciation of sex‐based differences in brain functionality.

## 2 METHODS

This systematic review was conducted based on the preferred reporting items for systematic reviews and meta‐analyses (PRISMA) (Liberati et al., 2009). The protocol of this review was registered on the International Prospective Register of Systematic Reviews (PROSPERO) website (Registration No. CRD42022298892).

### 2.1 Search strategy

PubMed and Scopus were searched using a combination of keywords in relation to Schizophrenia, fMRI, and sex differences, which are described in Supplementary Table S1. We did a search update until August 17, 2022. Two authors independently screened the available literature, by searching Pubmed and Scopus databases, using the title and abstracts to identify potentially eligible studies. Full‐texts of these studies were then reviewed for relevance to our aims. Reference lists of included studies were also screened to find any other potentially suitable studies. Any disagreements in the screening process were resolved through discussion.

### 2.2 Study selection and retrieval

Observational studies (case–control, cohort, and cross‐sectional) that used fMRI to evaluate neuroimaging differences in schizophrenia and assessed the effects of sex, were included in the present systematic review. There were no limitations regarding the activity completed during scanning (resting vs. task‐based), language, and publication date. The criteria for excluding studies were as follows: (1) Studies that did not evaluate brain differences using fMRI; (2) Studies that did not evaluate patients with schizophrenia; (3) Studies that did not specifically evaluate sex differences; (4) Literature reviews, books chapters without unique experimental data included, opinions, letters, and conference abstracts; (5) In vitro and nonhuman studies.

### 2.3 Data extraction

The following data was extracted from included studies: first author name, date of publication, the sample size and sex ratio, participant medications, age, the type of fMRI used in the evaluation of participants (task‐based vs. resting state), matched covariates, handedness, race distributions, and fMRI findings. The data were extracted by two independent reviewers, and disagreements were resolved through discussion.

### 2.4 Risk of bias assessment

The quality of included studies was assessed using the Newcastle–Ottawa scale (NOS), which screens the quality of data regarding selection, comparability, and exposure (Stang, 2010). Using this tool, a maximum score of eight can be achieved for each case–control study. Besides that, we used specific criteria published by Viswanathan et al. to evaluate the risk of bias for included studies (Viswanathan et al., 2008). Two authors independently assessed the included studies regarding their quality and risk of bias and discrepancies were resolved through discussion.

## 3 RESULTS

### 3.1 Summary of overviewed studies

**FIGURE 1:**
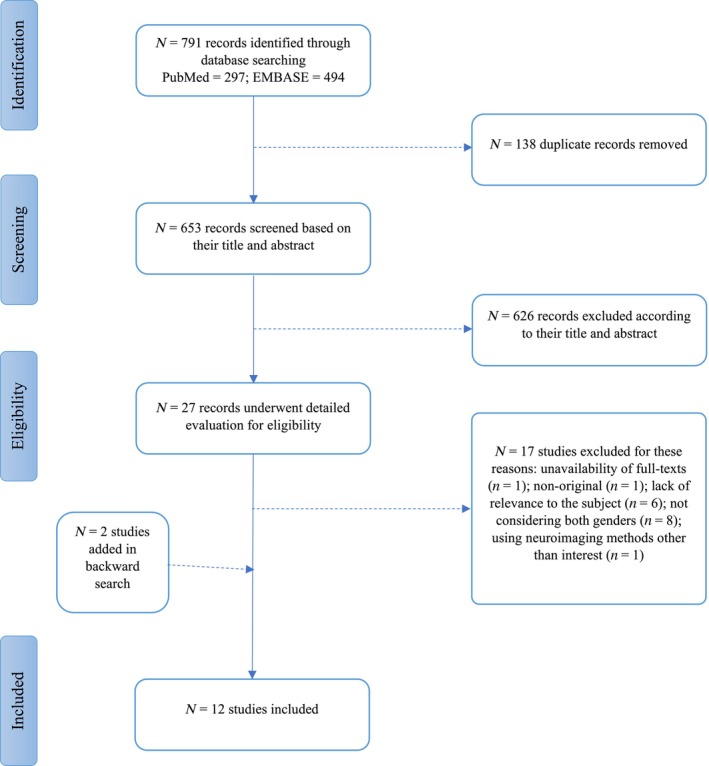
Flowchart of study selection.

Our initial search of two major databases resulted in a total of 791 articles (297 search results by PubMed, and 494 by Scopus). After removing duplicates (*n* = 138), the remaining 653 articles were screened for abstracts and titles, which resulted in the exclusion of 626 studies. After full‐text assessment, 17 additional papers were excluded due to: the population under study was not schizophrenia (*n* = 6) (Anderson et al., 2013; Brébion et al., 2021; Coman et al., 2010; Garcia et al., 2020; Sen & Parhi, 2019; Sokhi et al., 2005); evaluating only one sex (*n* = 8) (Abel et al., 2003; Das et al., 2012; Kircher et al., 2008; Kubicki et al., 2003; Kumari et al., 2009; Sommer et al., 2003; Zhang et al., 2021; Zhou et al., 2016); not being an original paper (*n* = 1) (Mendrek, 2013); not reporting fMRI findings (*n* = 1) (Bang et al., 2019); no full‐texts available (*n* = 1) (Mendrek, 2007). By screening reference lists of the included articles, we also identified two additional articles (Iraji et al., 2022; Lakis & Mendrek, 2013) not detected in the literature search using our a priori keywords. Following the above extraction process, 12 original papers were included in this study. Supplementary information about the inclusion and exclusion process is provided in Figure 1.

### 3.2 Overview

In this systematic review, 12 case‐controlled studies were included (Elsabagh et al., 2009; Iraji et al., 2022; Jiménez et al., 2010; Lakis & Mendrek, 2013; Lei et al., 2015; Ma et al., 2016; Mendrek et al., 2007; Mendrek, Jiménez, et al., 2011; Mendrek, Lakis, & Jiménez, 2011; Miller et al., 2015; Sommer et al., 2003; Wang et al., 2019). These studies were published between 2003 and 2022. The included studies investigated sex‐related neuroimaging findings in schizophrenia by task‐based fMRI (Elsabagh et al., 2009; Jiménez et al., 2010; Lakis & Mendrek, 2013; Mendrek et al., 2007; Mendrek, Jiménez, et al., 2011;Mendrek, Lakis, & Jiménez, 2011; Sommer et al., 2003) and resting‐state fMRI (Iraji et al., 2022; Lei et al., 2015; Ma et al., 2016; Miller et al., 2015; Wang et al., 2019). All extracted studies had recruited schizophrenia patients as the case group and healthy, non‐affected individuals as the control group, except for two that did not include a healthy control group but, instead, specifically evaluated for sex differences only in individuals diagnosed with schizophrenia (Mendrek et al., 2007; Mendrek, Jiménez, et al., 2011). In the majority of included studies, the number of female schizophrenia patients was less than males except for two having equal numbers (Mendrek, Lakis, & Jiménez, 2011; Sommer et al., 2003), and two with more females (Lei et al., 2015; Ma et al., 2016). Participants were similarly matched for sex in five studies (Elsabagh et al., 2009; Lakis & Mendrek, 2013; Lei et al., 2015; Miller et al., 2015; Wang et al., 2019), for age in five studies (Jiménez et al., 2010; Lakis & Mendrek, 2013; Lei et al., 2015; Mendrek, Lakis, & Jiménez, 2011; Miller et al., 2015), for parental socioeconomic status in three studies (Jiménez et al., 2010; Lakis & Mendrek, 2013; Mendrek, Lakis, & Jiménez, 2011), for handedness in three studies (Lakis & Mendrek, 2013; Mendrek, Lakis, & Jiménez, 2011; Miller et al., 2015), and for race distributions in only one study (Miller et al., 2015). Several studies evaluated for associations with disease duration, which was in the range of 5–117.5 months (Elsabagh et al., 2009; Jiménez et al., 2010; Ma et al., 2016; Mendrek et al., 2007; Mendrek, Jiménez, et al., 2011; Mendrek, Lakis, & Jiménez, 2011; Miller et al., 2015). In all included studies, patients were maintained on prescribed antipsychotic medications. Detailed characteristics of the included studies are provided in Table 1.

**TABLE 1:** Characteristics of included studies.

Study	Study design	Study groups	Sample size (F/M)	Age (years) (mean ± SD) (F/M)	Type of fMRI	Duration of disease (F/M)	Medication	Matched for
Sommer et al., 2003	Case–control	Schizophrenia	12/12^a^	33.6 ± 8/27 ± 6^a^	Task based fMRI	–	Atypical antipsychotic (six patients used clozapine [mean dose 339 mg, SD 67], five patients used olanzapine [all 15 mg] and one patient used risperidone [3 mg]). Oral‐contraconceptives in five patients.	–
		Healthy control	12/12^a^	32 ± 12/28 ± 5^a^				
Mendrek et al., 2007	Case–control	Schizophrenia	10/15	28.4 (±9.1)/26.4 (±5.8)	Task based fMRI	5 ± 3 years	Antipsychotic medication (four patients received one, eight received two, one received three, and one received four antipsychotics: haloperidol, *n* = 4, mean dosage 7.59/2.9 mg; zuclopentixol, *n* = 4, mean dosage 137.5 mg 9/12.5; risperidone, *n* = 12, mean dosage 4.09/2.4 mg; olanzapine, *n* = 8, mean dosage 20.09/4.6 mg; and/or quetiapine, *n* = 12, mean dosage 470.89/182.7 mg).	–
Elsabagh et al., 2009	Case–control	Schizophrenia	10/15	44.80 (±9.68)/41.00 (±9.97)	Task based fMRI	21.60 (±11.47)/16.13 (±12.31) years	Typical antipsychotics. (The patient sample was restricted to those on typical antipsychotics for 6 weeks or longer because atypical antipsychotics are known to improve frontal lobe responses.)	Sex
		Healthy control	10/15	36.10 (±14.38)/41.33 (±7.33)				
Jiménez et al., 2010	Case–control	Schizophrenia	17/16	32.11 (±7.76)/33.17 (±7.72)	Task based fMRI	12.88 (±7.82)/10.66 (±7.55) years	Atypical antipsychotic (clozapine, olanzapine, risperidone, quetiapine) (25 patients received one, 7 received two, 1 received three; clozapine: *n* = 19, mean dosage = 452.63 mg ± 77.23 mg; olanzapine: *n* = 11, mean dosage = 15 mg ± 5.6 mg; risperidone: *n* = 8 mean dosage = 4.00 ± 1.85 mg; quetiapine: *n* = 5, mean dosage = 550.00 mg ± 277.82 mg)	Age Parental socioeconomic status
		Healthy control	17/18	32.38 (±7.69)/31.64 (±6.50)				
Mendrek, Jiménez, et al., 2011	Case–control	Schizophrenia	10/15	28.40 (±9.17)/26.40 (±5.84)	Task based fMRI	5 ± 3 years	All patients were receiving treatment with at least one antipsychotic medication (four patients received one, eight patients received two, one patient received three, and one patient received four antipsychotics; haloperidol: *n* = 4, mean dosage = 7.5 ± 2.9 mg; zuclopentixol: *n* = 4, mean dosage = 137.5 mg ± 12.5; risperidone: *n* = 12, mean dosage = 4.0 ± 2.4 mg; olanzapine	–
Mendrek, Lakis, & Jiménez, 2011	Case–control	Schizophrenia	21/21	32.52 (±6.40)/31.71 (±7.43)	Task based fMRI	6.88 (5.44)/12.33 (8.02) years	Atypical antipsychotic (clozapine, olanzapine, risperidone, quetiapine) (30 patients received one, 11 received two, 1 received three; clozapine: *n* = 21, mean dosage = 452.63 ± 77.23 mg; olanzapine: *n* = 15, mean dosage = 5.00 ± 5.6 mg; risperidone: *n* = 13 mean dosage = 4.00 ± 1.85 mg; quetiapine: *n* = 9, mean dosage = 550.00 mg ± 277.82 mg)	Age Handedness Parental socioeconomic status
		Healthy control	21/21	29.08 (±9.19)/31.14 (±7.92)				
Lakis & Mendrek, 2013	Case–control	Schizophrenia	18/19	32.46 ± 7.66	Task based fMRI	–	All the patients received at least one atypical antipsychotic (chlorpromazine equivalence was calculated) (27 patients received one, 9 received two, and 1 received three. Clozapine: 𝑛 = 19, mean dosage = 452.63 [77.23] mg; olanzapine: 𝑛 = 12, mean dosage = 14.58 [5.4] mg; risperidone: 𝑛 = 11, mean dosage = 3.73 [1.67] mg; quetiapine: 𝑛=7, mean dosage = 585.71 [238.85] mg).	Age Sex Handedness Parental socioeconomic status
		Healthy control	18/19	31.81 ± 6.91				
Miller et al., 2015	Case–control	Schizophrenia	33/94	38.50 ± 11.83	Resting‐state fMRI	Minimally 1 year	All patients were clinically stable on antipsychotic medication for at least 2 months.	Age Sex Handedness Race distributions
		Healthy control	39/96	37.54 ± 11.27				
Lei et al., 2015	Case–control	Schizophrenia	63/61	24.62 (6.82)/24.31 (6.57)	Resting‐state fMRI	–	28 patients treated with low dose antipsychotics (risperidone or olanzapine; 25–75 mg of chlorpromazine daily dose equivalent) for less than 3 days prior to MRI scanning, the remaining patients (77.42%) were neuroleptic‐naive before scanning.	Age Sex
		Healthy control	52/50	24.71 (6.98)/24.80 (6.74)				
Ma et al., 2016	Case–control	Schizophrenia	44/51	34.9 (±10.5)/32.4 (±6.1)	Resting‐state fMRI	117.5 ± 93.0 months	Antipsychotic drugs: 85 patients were receiving medications at the time of the MRI examinations and 8 patients had never received any medications.	–
		Healthy control	56/43	36.3 (±11.5)/30.9 (±9.7)				
Wang et al., 2019	Case–control	Schizophrenia	131/186	–^b^	Resting‐state fMRI	–^b^	20 cases used Abilify, 8 cases used ZyprexaZydis, 5 cases used Risperdal, 5 cases used Prozac, 4 cases used Zyprexa, 4 cases used Invega, 4 cases used Solian, 4 cases used Flurazin, 4 cases used Seroquel, 4 cases used Dogmatyl, 3 cases used Seroxat, 2 cases used Zoloft, and 2 cases used other drugs.	Age Sex
		Healthy control	209/248	–^b^				
Iraji et al., 2022	Case–control	Schizophrenia	96/254	^c^	Resting‐state fMRI	–	–	–
		Healthy control	213/264	^c^				

Abbreviations: fMRI, functional magnetic resonance imaging; MRI, magnetic resonance imaging.

^a^ Data of men and control group are taken from the following article: Sommer I, Ramsey N, Kahn R. Language lateralization in schizophrenia, an fMRI study. *Schizophrenia Research*. 2001; 52 (1, 2): 57–67.

^b^ Age and duration of illness was reported for patients of the different sites in the article, but no single number was reported for all the participants.

^c^ Age of illness was reported for patients of the different sites in the article, but no single number was reported for all the participants.

### 3.3 Quality of the included studies

Table 2 reports the results of quality assessment for included studies using NOS. Each of the included studies was evaluated based on the selection of cases, comparability, and exposure. Studies were given a maximum score of eight, with a maximum score of four, two, and two, respectively, for each domain. Of the 12 included studies, eight received a score of five to six points, which reflected an overall good scientific quality (Iraji et al., 2022; Jiménez et al., 2010; Lakis & Mendrek, 2013; Lei et al., 2015; Ma et al., 2016; Mendrek, Jiménez, et al., 2011; Miller et al., 2015; Wang et al., 2019). There was a low level of risk of bias among all studies, as all studies applied inclusion/exclusion criteria, ruled out any impact from concurrent intervention or an unlimited exposure, handled the missing data properly, implemented valid and reliable measures for interventions/exposure and outcomes and, confounding variables. Moreover, apart from three studies (Ma et al., 2016; Mendrek et al., 2007; Sommer et al., 2003), confounding and modifying variables were accounted for in the experimental design. In none of the included studies were outcome raters blinded to diagnosis. Half of the included studies reported all pre‐specified outcomes (Jiménez et al., 2010; Lakis & Mendrek, 2013; Ma et al., 2016; Mendrek, Lakis, & Jiménez, 2011; Miller et al., 2015; Sommer et al., 2003). The results of the risk of bias assessment are provided in Table 3.

**TABLE 2:** Quality assessment of the included case–control studies.

	Selection					Comparability			Exposure			Score
Study	Case definition adequacy	Representativeness of the cases	Selection of controls	Definition of controls	Subtotal	Age	Sex	Subtotal	Ascertainment of exposure	Non‐response rate	Subtotal	
Sommer et al., 2003		*	*		2			0	*	*	2	4
Mendrek et al., 2007		*			1			0	*	*	2	3
Elsabagh et al., 2009		*			1		*	1	*	*	2	4
Jiménez et al., 2010	*	*		*	3	*		1	*	*	2	6
Mendrek, Jiménez, et al., 2011	*	*		*	3			0	*	*	2	5
Mendrek, Lakis, & Jiménez, 2011		*			1	*		1	*	*	2	4
Lakis & Mendrek, 2013		*			1	*	*	2	*	*	2	5
Miller et al., 2015		*			1	*	*	2	*	*	2	5
Lei et al., 2015		*	*		2	*	*	2	*	*	2	6
Ma et al., 2016	*	*		*	3			0	*	*	2	5
Wang et al., 2019		*	*		2	*	*	2	*	*	2	6
Iraji et al., 2022	*	*	*	*	4			0	*	*	2	6

* denotes the scoring

**TABLE 3:** Publication bias.

	Selection bias		Performance bias	Attrition bias	Detection bias				Reporting bias
Study	Q1	Q2	Q3	Q4	Q5	Q6	Q7	Q8	Q9
Sommer et al., 2003	Yes	No	Yes	Yes	No	Yes	Yes	Yes	No
Mendrek et al., 2007	Yes	No	Yes	Yes	No	Yes	Yes	Yes	Yes
Elsabagh et al., 2009	Yes	Yes	Yes	Yes	No	Yes	Yes	Yes	Yes
Jiménez et al., 2010	Yes	Yes	Yes	Yes	No	Yes	Yes	Yes	No
Mendrek, Jiménez, et al., 2011	Yes	Yes	Yes	Yes	No	Yes	Yes	Yes	Yes
Mendrek, Lakis, & Jiménez, 2011	Yes	Yes	Yes	Yes	No	Yes	Yes	Yes	No
Lakis & Mendrek, 2013	Yes	Yes	Yes	Yes	No	Yes	Yes	Yes	No
Miller et al., 2015	Yes	Yes	Yes	Yes	No	Yes	Yes	Yes	No
Lei et al., 2015	Yes	Yes	Yes	Yes	No	Yes	Yes	Yes	Yes
Ma et al., 2016	Yes	No	Yes	Yes	No	Yes	Yes	Yes	No
Wang et al., 2019	Yes	Yes	Yes	Yes	No	Yes	Yes	Yes	Yes
Iraji et al., 2022	Yes	Yes	Yes	Yes	No	Yes	Yes	Yes	Yes

### 3.4 Task‐based fMRI studies

Six studies observed sex‐based functional neuroimaging differences in schizophrenia patients, as revealed by task‐based fMRI using the BOLD modality (Table 4). Utilized tasks measured emotion response, visuospatial recognition, response inhibition, and semantic cognition.

**TABLE 4:** Sex differences findings in task based fMRI studies.

Study	Tesla sequence	Brain areas	Connectivity/activation	Task	Methods for sex differences analyses			Covariates	Results	FMRI correlated with neuropsychological test performance
					Sex by diagnosis effects					
					Model	Tested?	Findings			
Sommer et al., 2003	1.5 T	Volumes of interest: Brodmann areas (BAs) 44 and 45 (Broca's area and its contralateral homologue), middle temporal gyrus (BA 21), superior temporal gyrus (BA 22, 38, 41, 42 and 52), supramarginal gyrus (BA 40) and angular gyrus (BA 39).	Activation	1. The verb‐generation task (Every 3.6 s a name appears on the screen. The subject should create the appropriate verb for that word.) 2. The reverse‐read semantic decision task The subject should read the words that were spelled from right to left in a non‐mirror way. Words were displayed every 3.6 s. The subject was instructed to say the word silently if it was an animal and press a button. Both tasks were performed during 10 courses of 29 s. They rested for 29 s each time.	GLM	Yes	No	–	There were no differences between the two groups of male and female patients. In both cases, the lateralization was lower than the controls. The reason is the increased activation of the right‐hemispheric language and this shows a failure to control non‐dominant language areas in schizophrenia.	–
Mendrek et al., 2007	1.5 T	Whole brain	Activation	Negative and neutral conditions: Negative condition included 44 emotionally negative images (e.g., plane crash, angry face, electric chair) and neutral included 44 emotionally neutral images (e.g., boat, neutral face, chair). 4 blocks of neutral and four blocks of negative images were presented to the subjects. (They separated with a rest time of 14.4 s.) Subjects looked carefully at the images and gave their mental responses to the stimuli on a scale of 0 (no emotional response) to 8 (strongest emotional response).	GLM	Yes	Yes	–	Both sexes did not have significant differences in the intensity of subjective responses to aversive photos. Men showed greater cerebral activations than women in cerebellum, bilateral thalamus, temporal gyrus, left cuneus, right occipital gyrus and posterior cingulate, Women showed greater cerebral activation than men in the left middle frontal gyrus. Difference between men and women during passive looking of aversive images: Men activated bilateral hippocampal and left middle temporal gyrus, bilateral lingual and right inferior frontal cortex, bilateral precuneus and cerebellum. Women activated left lingual, right inferior frontal, left middle occipital cortices, and bilateral cerebellum.	–
Elsabagh et al., 2009	1.5 T	Whole brain	Activation	N‐back task: Participants observed the positions of dots, which appeared at one of the 4 corners of a box in a random instruction. There were 3 situations (0‐, 1‐, and 2‐back). Each position was presented five times in a pseudo‐random instruction and lasted 30 s. Members had to press the button corresponding to the right position of the current (0‐back) or previously presented (1 back = previous screen, 2‐back = previous screen plus one) stimulus. 15 stimuli were displayed in each 30 s active block. Each active condition began with a 15 s rest condition. In total, this task lasted 11 min and 15 s.	GLM	Yes	Yes	Age, duration of illness	In Female patients: 0‐back > rest. Negative correlation between a large cluster with maximal activity in the left lower frontal region and disease duration. 1‐back >0‐back. Negative correlation between right cerebellum activity at the uncorrected level and duration of disease. 2‐back >0 back. Negative significant correlation between the activity of a large cluster (including right superior temporal gyrus, and the left and right cerebellum) and the duration of the disease. In male patients: 0‐back > rest. Negative relationship between activity in the left parietal lobe and duration of disease 1‐back >0‐back. No relationship was shown. 2‐back >0 back. Negative relationship between a cluster (located in the dorsolateral prefrontal cortex) and the duration of the disease.	Decreased response accuracy was observed with increasing memory load in both groups. Patients' performance was significantly worse than the control group. As the load increased, the patients efficacy decreased more than the control group. There were no major sexual effects on performance. Performance accuracy in each of the 3 tasks was not related to the duration of the disease. Performance accuracy was not related to age in the patient group.
Jiménez et al., 2010	3 T	Whole Brain	Activation	8‐min run of alternating 38‐s blocks of experimental and control conditions with 20‐s periods of rest separating the blocks from one another. Presentations of pairs of black‐and‐white drawings of 3‐D shapes. In the experimental condition, one shape was rotated along its vertical axis relative to the other shape. In half of the trials, the figures were identical to each other, whereas in the other half they were mirror images of each other. In the control condition, participants were presented with the unrotated identical or mirror 3‐D drawings. In both conditions participants had to determine (by pressing a button with their right index or middle finger) whether the two shapes were identical or mirror images of each other. Each picture appeared for duration of 3000 ms followed by a blank screen with a fixation point for an average of 1.75 s (ranging from 1 to 2.5 s and giving an average inter stimulus interval [ISI] of 4.75 s).	GLM	Yes	Yes	–	Significant activations in the right precuneus and prefrontal cortex (superior, middle and medial gyrus) in schizophrenia men (No significant deactivations). Significant activations in the parietal cortex (superior, inferior and precuneus), the inferior frontal cortex and in the right middle occipital cortex, also relative deactivations in the superior frontal cortex in Schizophrenia women.	Only in women, a positive correlation was observed between activation and functional accuracy in the right fusiform gyrus.
Mendrek, Jiménez, et al., 2011	1.5 T	Whole brain—regions of interest (ROI): hippocampus, para hippocampus, amygdala, superior temporal gyrus, orbitofrontal cortex, and cingulate gyri, bilaterally	Activation	Showing one block of emotionally sad condition (e.g., death of a beloved person such as parents or a friend) and one block of neutral condition (e.g., various human activity such as interviews, carpentry, gardening, etc.). They separated with a rest period (blue cyan screen). Sad and neutral conditions were matched with the sad film excerpts in respect to the number and the gender of the individuals involved.	GLM	Yes	Yes	Positive and negative symptom scores, the medication dose (chlorpromazine equivalence)	In male patients: There were significant correlations between negative symptoms and activations in several cortical and subcortical limbic regions including Region of Interests in: right middle frontal, bilateral superior frontal, left superior temporal and cingulate cortices. Search of the whole brain also showed significant correlations in left cerebellum, bilateral caudate, and right precentral gurus. There were no associations with positive symptoms. In female patients: There were opposite correlations between positive symptoms and activation in the left middle temporal, right hippocampus, right superior temporal, and left middle frontal gyrus. Search of the whole brain also showed significant correlations in the right lingual, right middle occipital, and left postcentral. There were no associations with negative symptoms.	‐
Mendrek, Lakis, & Jiménez, 2011	3 T	Whole Brain	Activation	Mental rotation task involved presentations of pairs of black and white drawings of 3‐D shapes. We used a block‐design where blocks of experimental and control conditions were repeated four times during the course of the functional MRI run. In the experimental condition one shape was rotated along its vertical axis relative to the other shape. In half of the trials, figures were identical, whereas in the other half they were mirror images. In the control condition, participants were presented with the unrotated identical or mirror 3D drawings. In both conditions participants had to determine whether the two shapes were identical or mirror images of each other.	GLM	Yes	Yes	–	In women with schizophrenia, a similar pattern of extensive brain activity was showed in the parietal and lateral prefrontal cortex, but men with schizophrenia revealed much more limited activity in the prefrontal cortex. A positive correlation was observed between testosterone levels and activation of the large cluster of right middle frontal cortex and right cerebellum in schizophrenic women. In schizophrenia men, no significant correlations were found. In women with schizophrenia, the correlation analysis between data from fMRI and estradiol showed a significant correlation in the right Para hippocampal gyrus.	–
Lakis & Mendrek, 2013	3 T	Whole brain	Activation	Viewing blocks (positive, negative, and neutral pictures while in the fMRI scanner) Each block was 48.5 s in length and there were 16‐s periods of rest separating the blocks from one another.	GLM	Yes	Yes	–	Men with schizophrenia had an increased response to neutrally emotional content. Activated brain region in schizophrenia men: Right Middle cingulate gyrus, Left Superior frontal gyrus, Left Inferior parietal gyrus, Right Inferior parietal gyrus, Left Supplementary motor area, Left Precuneus, Right Anterior cingulate gyrus, Left& Right Cerebellum, Right Precentral gyrus, Right Middle frontal gyrus, Right Superior orbitofrontal cortex, Left Middle temporal gyrus, Left Supramarginal gyrus. Activated brain region in schizophrenia women: Left Putamen, Right Angular gyrus.	–

Abbreviations: FC, functional connectivity; fMRI, functional magnetic resonance imaging; GLM, general linear model; Rs, resting state; T, Tesla.

#### 3.4.1 Emotion response tasks

**FIGURE 2:**
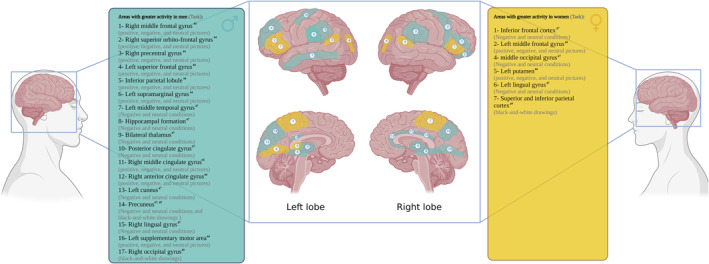
An overview of sex differences in brain function in task‐based fMRI studies. Created with Biorender.com.

Three studies that presented randomized image blocks designed to induce positive, negative, and neutral emotions demonstrated greater activation of the cerebellum for males with schizophrenia than for female patients regarding negative emotions (Mendrek et al., 2007). Another study, which alternated one block of emotion‐inducing images and one block of neutral images, reported a positive correlation between negative symptoms and cerebellum activation in male, but not female, participants with schizophrenia (Mendrek, Jiménez, et al., 2011). Moreover, randomized, alternating presentation of positive, negative, and neutral pictures in a third study demonstrated that males with schizophrenia showed more activation in the cerebellum in response to emotionally neutral stimuli (Lakis & Mendrek, 2013). Two of these studies found that males with schizophrenia showed more activation in the temporal gyrus than females in response to emotionally upsetting images, such as the death of a parent or friend (Mendrek et al., 2007; Mendrek, Jiménez, et al., 2011). Additionally, a third study demonstrated that males with schizophrenia, but not females, had increased temporal gyrus activation in response to emotionally neutral faces (Lakis & Mendrek, 2013). This study also found that male patients, but not female patients, had activation of subregions of the cingulate gyrus in response to neutral faces, as well as increased activation of the parietal lobes (Lakis & Mendrek, 2013). Furthermore, some of the included studies demonstrated a negative correlation between the severity of negative symptoms and reduced activation of the cingulate gyrus to neutral faces for males with schizophrenia (Mendrek et al., 2007; Mendrek, Jiménez, et al., 2011). Occipital lobe activation, in one study, showed differential hemispheric patterns of response between male and female patients with schizophrenia, wherein males showed increased right occipital activation while females showed increased activation in the left occipital lobe (Mendrek et al., 2007). In another study, there was a positive correlation between bilateral right middle occipital cortex activation and positive symptoms only in females diagnosed with schizophrenia (Mendrek, Jiménez, et al., 2011). Considering the frontal lobes, there was substantial heterogeneity in activation responses between studies. For instance, one study showed that, in contrast to the activation of the superior region of the frontal cortex in male patients in response to neutral emotional stimuli, the inferior frontal cortex was activated in female patients (Lakis & Mendrek, 2013). Another study demonstrated a strong relationship between negative symptoms and activation of the right middle frontal region in males but an opposite correlation between positive symptoms and activation of the left middle frontal region in females (Mendrek, Jiménez, et al., 2011). Moreover, one of the studies showed no significant sex‐related differences in frontal lobe activity (Mendrek et al., 2007) (Figure 2).

#### 3.4.2 Visuospatial recognition tasks

Two of the included studies of participants with schizophrenia used the mental rotation task, in which pairs of black and white drawings of 3‐D shapes are presented to participants, to evaluate sex differences in fMRI BOLD responses related to visuospatial recognition and manipulation of objects. These studies demonstrated increased activations in the parietal cortex of females with schizophrenia (Jiménez et al., 2010; Mendrek, Lakis, & Jiménez, 2011). Moreover, those studies reported that female patients also showed more activation in the prefrontal lobe compared to male patients (Jiménez et al., 2010; Mendrek, Lakis, & Jiménez, 2011). Additionally, one of the studies showed strong activation in the right precuneus cortex only for males with schizophrenia (Jiménez et al., 2010). The other study found a positive correlation between levels of testosterone and activation of the frontal cortex and the cerebellum in female patients, but no similar correlations were observed in males with schizophrenia (Mendrek, Lakis, & Jiménez, 2011). That study also demonstrated a positive correlation between estradiol levels and activation of the parahippocampal gyrus in female patients (Mendrek, Lakis, & Jiménez, 2011) (Figure 2).

#### 3.4.3 Response inhibition tasks

One study used the N‐back task to evaluate for sex differences in schizophrenia. In this task, participants have to determine the matching of each stimulus, a presentation of dot arrays, with one that appeared either 0, 1, or 2 items earlier. This study demonstrated that for female patients, disease duration was negatively correlated with activation of the cerebellum, frontal cortex, and temporal gyrus, but did not specify the hemispheric lateralization. In contrast, males with schizophrenia showed a negative correlation between duration of illness and parietal and prefrontal lobe activation, with no hemispheric lateralization specified in the paper (Elsabagh et al., 2009).

#### 3.4.4 Semantic cognition tasks

Using verb‐generating and reverse‐read semantic decision tasks, one of the included studies observed no significant sex differences in participants with schizophrenia (Sommer et al., 2003).

### 3.5 Resting‐state fMRI studies

Five studies used resting‐state fMRI, employing a whole‐brain approach, to evaluate sex differences in schizophrenia patients. These studies used arterial spin labeling (ASL), amplitude of low‐frequency fluctuations (ALFF), and functional connectivity modalities as three main analytic approaches to assess for sex differences among these patients. Experimental details and neuroimaging findings of these studies are provided in Table 5.

**TABLE 5:** Sex differences findings in resting‐state fMRI studies.

Study	Tesla sequence	Brain areas	Connectivity/activation	Methods for sex differences analyses			Covariates	Results	FMRI correlated with neuropsychological test performance
				Sex by diagnosis effects					
				Model	Tested?	Findings			
Miller et al., 2015	3 T	Whole Brain	Activation	GLM	Yes	Yes	–	In men, midrange spatial frequencies were predominant over temporal frequency. In women, high spatial frequencies prevailed over most temporal frequencies, and in temporal frequencies greater than 0.10 Hz, all spatial frequencies were predominant, except for midrange.	–
Lei et al., 2015	3 T	Whole brain Region of interest (bilateral putamen, inferior parietal lobe, posterior cingulate cortex and middle temporal gyrus)	Activation	GLM	Yes	Yes	Age Years of education	In male patients, a significant decrease in degree centrality occurred in the right putamen but in females in the middle frontal gyrus. Males also showed a higher range of low‐frequency fluctuations in the frontal and parietal regions, insula, lingual gyrus and cerebellar tonsil than females. Males also had lower amplitude of low‐frequency fluctuations in the cerebellar posterior lobe, lentiform nucleus, Hippocampus and Para Hippocampal compared than females.	–
Ma et al., 2016	3 T	Whole brain	Activation	GLM	Yes	Yes	–	Men with schizophrenia have more blood flow in the frontal regions of the brain than women, and relatively less cerebral blood flow in the posterior regions of the brain. Just the left middle frontal gyrus had a specific gender difference in amplitude of low frequency fluctuations. Females had more brain areas with diagnostic group differences than males.	–
Wang et al., 2019	–	Whole brain	Rs‐FC	GLM	Yes	Yes	The mean displacements after scrubbing	**Lateralization of Functional Connectivity Changes in First‐Episode Schizophrenia:** Functional changes were more common in men with schizophrenia in the left hemisphere than in the right hemisphere, but more common in women with schizophrenia in the right hemisphere. Men, unlike women, had significant changes in the connection to the Broca area in the left hemisphere. Compared to men, changes were more spread in females, such as: structures on the medial and orbital frontal and lateral surfaces. Specifically, the only node in both men and women that changes during the first episode is the orbital part of the inferior frontal gyrus. **Lateralization of Functional Connectivity Changes in Chronic Schizophrenia** In both sexes, functional changes for patients spread posteriorly. Both also showed special changes in thalamo‐cortical junctions. Males who were lateralized to the left in the first episode were laterally to the right in the chronic phase, but the female patients were still lateralized to the right. In males, functional changes in the temporal lobe occurred bilaterally in connection with the precentral gyrus. In females, bilateral functional changes in the olfactory pathways occurred in association with anterior and dorsal cingulate gyrus and precentral gyrus on both sides. **Sex‐Specific Laterality Patterns of Functional Connectivity Change** In first episode male patients, none of the connections are limited to the right hemisphere, but in female patients half of the connections are correlated with the right hemisphere. (Just 2 of 19 connections are among structures both located in the left hemisphere.) In the chronic stage, both sexes displayed right lateralization in functional connectivity changes, however female patients showed a higher level of right lateralization.	In the first‐episode in male patients functional connectivity between left inferior triangular and right inferior opercular frontal gyrus was related to delusions. In females, connectivity between the posterior cingulate gyrus and the left inferior orbital frontal gyrus associated with poor impulse control and hostility. In the chronic state, both sexes showed a significant association with thalamic involvement. In the cortex, there was a significant correlation between symptom score and the right hemisphere. In men, this association was with right middle frontal or temporal gyrus, but in women with right pre and postcentral gyrus.
Iraji et al., 2022	3 T	Whole Brain	Rs‐FC	GLM	Yes	Yes	Age Data acquisition site Mean frame‐wise displacement Symptom score	(1) Patients show decreased sFNC strength between and within the somatomotor and temporal amplitudes in both gender cohorts. The dFNC results showed that these differences occurred mainly in State 4 for women and State 3 for men. Sex differences in the somatomotor and temporal amplitudes are more obvious in dFNC states, exclusively in State 4. (2) Between cognitive control and default state, sFNC showed significant differences between men and women. (3) dFNC in male and female showed significant differences in State 4. In men, the power of dFNC decreased compared to the control group, but increased in women. (4) While gender differences present important effects of schizophrenia in males for functional amplitude connectivity associated with the somatomotor and the visual, the opposite pattern occurred for the rest of the differences. (One exception is the within temporal amplitude functional connectivity in the dFNC State 4 between model order 25 and 50.)	Significant sex differences observed among several functional amplitudes, including in temporal and subcortical connectivity patterns. This significantly correlates with symptom scores in only males but (but not females). Affected functional areas are predominantly altered in schizophrenia and are being touted as potential for biomarker identification.

Abbreviations: dFNC, dynamic functional network connectivity; FC, functional connectivity; fMRI, functional magnetic resonance imaging; FNC, functional network connectivity; GLM, general linear model; Rs, resting state; sFNC, static functional network connectivity; T, Tesla.

*Note*: State 3 demonstrates overall positive FNC across the cerebral cortex, potentially representing global functional integration. Of particular note, the cerebellum shows overall negative FNC with cerebral functional domains in State 3. The negative association between the cerebellar domain and sensorimotor functional domains is prominent in State 4. State 4 can be distinguished with strong functional integration between the visual, somatomotor, and temporal domains, and their anticorrelation patterns with the rest of the brain. This state also shows strong functional integration between the subcortical and cerebellar domains.

#### 3.5.1 ASL modality

One study evaluated temporal and spatial frequencies, demonstrating that, unlike female patients who showed a predominance of high spatial frequencies over most temporal frequencies, males with schizophrenia predominantly demonstrated midrange spatial frequencies over temporal frequencies (Miller et al., 2015).

#### 3.5.2 ALFF modality

**FIGURE 3:**
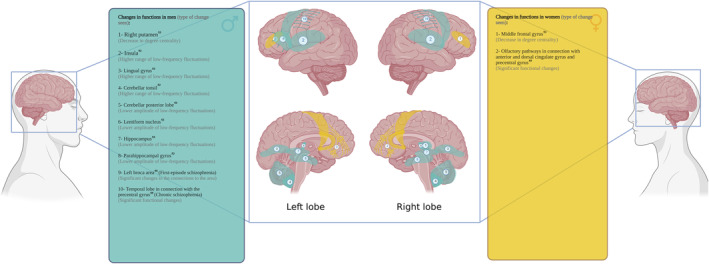
An overview of sex differences in brain function in resting state fMRI studies. Created with Biorender.com.

Two of the included studies evaluated low‐frequency fluctuations in patients with schizophrenia. One of those studies found that male patients showed a greater occurrence of low‐frequency fluctuations in frontal and parietal lobes and the insular cortex (the hemispheric lateralization not specified), whereas in female patients there was a higher range of low‐frequency fluctuations in the hippocampus, parahippocampus and lentiform nucleus (Lei et al., 2015). In contrast, the other study reported a higher amplitude of low‐frequency fluctuations in the left middle frontal gyrus in males compared to females with schizophrenia (Ma et al., 2016). Moreover, in that study, males with schizophrenia exhibited greater blood flow in the frontal regions (hemispheric lateralization not specified), whereas females with schizophrenia demonstrated greater blood flow in posterior regions (Ma et al., 2016) (Figure 3). Notably, these differences were also observed between male and female controls. However, differences between schizophrenia and healthy control groups were more prominent in females.

#### 3.5.3 BOLD functional connectivity modality

One rsfMRI study evaluated the lateralization of functional connectivity changes across different clinical phases of schizophrenia. The authors reported that at the onset of a schizophrenia diagnosis, most of the resting state fMRI network differences occurred in the left hemisphere (including Broca's region) in male patients, whereas female patients demonstrated primarily right hemispheric differences. During the chronic phase of schizophrenia, female patients in comparison to healthy female control subjects exhibited persistent lateralization of functional connectivity differences to the right hemisphere, whereas the pattern of altered functional connectivity in males with schizophrenia tended to lateralize toward the right hemisphere with greater duration of illness (Wang et al., 2019) (Figure 3).

An additional rsfMRI study evaluated static functional network connectivity (sFNC) differences, taking the assumption that FC does not change over data acquisition time periods of 2 s, and dynamic functional network connectivity (dFNC). It studied non‐stationary changes in FC during a data acquisition interval of 44 s and slide step size of 2 s. This study demonstrated that the somatomotor and temporal region amplitudes showed more obvious sex‐based differences in the dFNC states. Moreover, both males and females with schizophrenia showed significant disparities in sFNC between cognitive control and default states (Iraji et al., 2022).

## 4 DISCUSSION

Previous fMRI studies have reported sex‐based differences in various psychiatric disorders, such as bipolar disorder, major depressive disorder (MDD), and autism spectrum disorder (Jogia et al., 2012; Talishinsky et al., 2022; Walsh et al., 2021). However, to the best of our knowledge, this is the first systematic review to provide a comprehensive overview of fMRI studies investigating sex differences in schizophrenia. Reviewing task‐based fMRI studies, sex‐based differences in fMRI results appeared to predominantly involve the bilateral cerebellum, prefrontal lobe (hemispheric lateralization not specified), right middle frontal cortex, right cingulate cortex, bilateral precuneus, and left putamen regions of the brain. Moreover, resting‐state fMRI studies revealed differences between males and females in several brain regions, involving the frontal gyrus, temporal lobe, parietal cortex, insula, Broca region, and hippocampus, although any hemispheric laterality was not specified.

The prefrontal cortex, a brain region involved in memory and cognitive processes, is implicated in schizophrenia and other psychiatric disorders (Ranganath et al., 2008). The degeneration of neurons connecting the prefrontal cortex to the cerebellum has been postulated to be a primary factor influencing the severity of negative symptoms in patients with schizophrenia (Brady et al., 2019). Mendrek et al. showed increased brain activity of this region in females with schizophrenia who exhibited less severe negative symptoms compared to males having a similar duration of illness, which can suggest a compensatory mechanism in prefrontal cortex of women with schizophrenia, causing resilience against severe negative symptoms. In this study, a relationship between sex steroid hormones and cerebral function was proposed to explain this finding. In the context of testosterone levels being increased in female schizophrenia patients relative to same‐sex controls, whereas male patients with schizophrenia show decreased overall testosterone levels compared to healthy male controls, a positive correlation between upregulation in sex steroid hormone levels and cerebral activation may help to explain the increased prefrontal activity in women with schizophrenia that was found in this study. This study additionally demonstrated a positive relationship between activation levels of the prefrontal cortex and testosterone levels in healthy male controls (Mendrek, Lakis, & Jiménez, 2011). This finding may indicate that treatment with testosterone in the males with negative symptoms and low testosterone levels, may improve negative symptoms by increasing prefrontal cortex activation. In addition, other studies revealed that impairment of executive functioning may have a stronger effect on symptoms, especially negative symptoms, of male patients with schizophrenia than for female patients (Karilampi et al., 2011). Thus, sex‐based differences in symptom expression and regional brain relationships could, in part, reflect the effects of sex steroids that might be considered in developing new treatment targets. In this regards, further studies, comprising both larger sample sizes and a larger proportion of females, are required to more comprehensively investigate interactions between hormonal effects, symptom expression, regional brain relationships, and sex‐based differences in schizophrenia.

The cingulate cortex, a part of the limbic system involved in emotional regulation and memory, has been widely investigated for sex differences in psychiatric disorders. A recent MDD study indicated increased connectivity in the subgenual cingulate (hemispheric lateralization not specified) and posterior cingulate in males with MDD compared to HC males that was not found for females with MDD compared to HC female (Talishinsky et al., 2022). Subjects with schizophrenia are shown to have increased cingulate cortex activation in response to emotion‐eliciting stimuli compared to healthy controls (Rolls, 2015). Lakis et al. reported that patients with schizophrenia have increased activation in the right middle cingulate gyrus in response to neutral emotional content and that this increased activation is mainly observed in male patients (Lakis & Mendrek, 2013). Another study that also reported differential fMRI activation of the cingulate gyrus between males and females with schizophrenia found that males exhibited a lower level of activation when inhibiting responses to negative stimuli (Vercammen et al., 2013). In that same study, male schizophrenia patients showed an inverse correlation between serum testosterone level and activation of the bilateral middle frontal gyrus and left insula (Vercammen et al., 2013). Champagne and colleagues reported that increased levels of estradiol are correlated with reduced early selective attention processing, as well as reduced memory context updating and stimulus categorization. Those investigators also observed a significant positive correlation between testosterone levels and functions related to memory context updating (Champagne et al., 2014). Differences in cingulate gyrus functioning between male and female patients may reflect differential demands of cognitive processing in response to emotional tasks (Phan et al., 2002). This further highlights the need for systematic examination of sex interactions with hormonal effects, regional brain activation, and dysregulation of emotional processing in schizophrenia.

The temporal lobe, a part of the default mode network (DMN), is involved in episodic and working memory (Berron et al., 2020). Our review indicates that sex differences in schizophrenia may influence interactions between positive symptoms and activation of the temporal cortex. Wible et al. reported that during verbal memory tasks, females with schizophrenia, but not males, showed a negative correlation between temporal lobe activation and both positive and negative symptoms (Wible et al., 2009). Thus, in contrast to males with schizophrenia, an increase in the intensity of positive and negative symptoms appears to diminish temporal cortex activation to verbal memory tasks in females. Additionally, as an animal model of schizophrenia has shown that increased amounts of estradiol may reduce the risk of short‐term episodic memory loss (Cabeza et al., 2008), females with schizophrenia may have less short‐term episodic memory loss than male patients. Overall, the role of temporal lobe in memory formation is more prominent in females and affected by estradiol levels, suggesting a potential role for estradiol in improving memory loss in females with schizophrenia.

Task‐based fMRI studies of the parietal cortex, involved in memory retrieval and visuospatial processing (Karilampi et al., 2011), also demonstrate sex‐based differences in schizophrenia (Cabeza et al., 2008). Females with schizophrenia show decreased activation of the parietal cortex compared to males, which may reflect differences in visual task performance. In this regard, clinical trials using computerized assessments to assess fronto‐temporal system function have shown that females with schizophrenia have higher visual task processing speeds but less accuracy than males (Gur et al., 2001; Halari et al., 2006). Since accuracy is considered to be correlated with activation levels of the parietal cortex, we can conclude that activation of the parietal cortex is diminished in females with schizophrenia (Halari et al., 2005). Moreover, it seems that increased activation of the parietal lobe in male patients during the mental rotation task is correlated with their weaker behavioral performance compared to healthy males (Jiménez et al., 2010). Hence, future studies that sex‐match participants may help to address this confounding effect.

In addition, some of the reported sex‐based differences in different parts of the brain in patients with schizophrenia are shown also in healthy controls. Recent studies showed higher activation levels of the rostral anterior cingulate in healthy males compared to healthy females, which can explain behavioral differences between the two sexes (Liu et al., 2012). Evans and colleagues attempted to evaluate sex differences in the activation level of the prefrontal cortex among healthy adults. This study showed that during the reversal learning task, male adults reflect better performance in the reversal phase than females, which can indicate sex‐related differences in the activation level of the prefrontal cortex (Evans & Hampson, 2015). It is shown that language‐related areas of the brain, including the temporal lobe, in healthy females are more activated and expanded compared to males. Still, healthy males have better performances regarding visuospatial tasks, which can indicate higher activation levels in related areas, including the parietal cortex (Brun et al., 2009). Further studies evaluating sex differences in brain function in patients with schizophrenia should include healthy controls matched due to sex to provide more information on whether founded differences are related to sex‐disease interaction or just sex.

This review has several limitations that must be commented upon. First, some of the included studies reported small sample sizes and did not match participants for possible confounding variables, such as age, handedness, duration of illness, socioeconomic factors, or race, that varied substantially among participants and were not systematically examined for sex‐based differences. Second, pre‐study medications were maintained at enrollment in all included studies, but antipsychotic and other medication use, or equivalent dosage, was not accounted for in most of the reported studies. As male patients are typically prescribed higher doses of medications over longer durations, this may have influenced fMRI findings. Another important consideration is the exclusion of studies from this review that did not specifically evaluate sex differences. Although studies using fMRI to investigate schizophrenia may report findings in male and female subjects as a secondary outcome, we included only studies that were specifically designed to evaluate for sex‐based differences. This could have increased the potential bias for not identifying null findings, i.e., studies that did not observe any effect of sex as a covariate on fMRI results. A related limitation was the small number of female participants enrolled in most fMRI studies of schizophrenia, that precluded many or most of those studies from having sufficient statistical power to report meaningful negative findings. A further limitation reflects the growing recognition that many earlier rsfMRI studies, including the possibility of studies included in this review, did not collect sufficient data to account for the impact of subtle head movement artifacts on correlational modeling (Power et al., 2014). In this regard, the possibility of sex‐based differences in movement artifacts could further impact the interpretation of rsfMRI results. Finally, interpretation of fMRI findings from included studies relied on widely different acquisition parameters and study/task designs.

In conclusion, due to differences in brain morphometry and activation patterns observed between males and females in other clinical and experimental settings, we aimed to report on sex‐based differences in fMRI studies of schizophrenia. Findings to date, limited by small sample sizes and methodological shortcomings, suggest an interaction between diagnosis and sex‐based effects on fMRI responses. There are sex‐based differences in specific task‐induced regional brain activation patterns, involving the cerebellum, prefrontal lobe, right middle frontal cortex, cingulate cortex, precuneus, and left putamen brain regions, as well as resting state fMRI differences primarily observed in the frontal gyrus, temporal lobe, parietal cortex, insula, Broca area, and hippocampal regions. Future studies of schizophrenia that seek to systematically evaluate the interaction of sex and fMRI diagnostic differences will require larger sample sizes with more equal proportions of carefully matched male and female participants, as well as stratification based on sex, in order to shed further light on possible sex‐based brain differences in schizophrenia. The result will be an improved understanding of the pathophysiologic underpinnings and manifestations of schizophrenia that may allow improved targeting of treatment.

## CONFLICT OF INTEREST STATEMENT

The authors declare no conflicts of interest.

## Supporting information


**Table S1.** Search strategies for databases.

## Data Availability

The data that support the findings of this study are available from the corresponding author upon reasonable request.
