# Supplementary material for: Brain‐based sex differences in schizophrenia: A systematic review of fMRI studies
Source: Hum Brain Mapp. 2024 Mar 23;45(5):e26664. doi: 10.1002/hbm.26664 (PMC10960555; doi:10.1002/hbm.26664)
Supplement: Supplementary file 1 — Table S1. Search strategies for databases. [file HBM-45-e26664-s001.docx]

| **Table S1.** Search strategies for databases | |
| --- | --- |
| **Database** | **Search string** |
| PubMed | (("Phenotypic Sex"[Title] OR "Sex, Phenotypic"[Title] OR "Genotypic Sex"[Title] OR "Sex, Genotypic"[Title] OR "sex"[Title] OR "women"[Title] OR "female"[Title] OR "men"[Title] OR "male"[Title] OR "gender"[Title] OR "sex difference"[Title] OR "gender difference"[Title] OR “sex-specific”[Title] OR “sexual”[Title]) AND ("Schizophrenias" OR "Schizophrenic Disorders" OR "Disorder, Schizophrenic" OR "Disorders, Schizophrenic" OR "Schizophrenic Disorder" OR "Dementia Praecox" OR "schizophrenia")) AND (fMRI OR "functional magnetic resonance imaging" OR MRI OR "magnetic resonance imaging" OR "brain activity" OR "brain function" OR connectivity OR "neural activity") |
| Scopus | ( TITLE ( "Phenotypic Sex" OR "Sex, Phenotypic" OR "Genotypic Sex" OR "Sex, Genotypic" OR "sex" OR "women" OR "female" OR "men" OR "male" OR "gender" OR "sex difference" OR "gender difference" OR “sex-specific” OR “sexual”) AND TITLE ( "Schizophrenias" OR "Schizophrenic Disorders" OR "Disorder, Schizophrenic" OR "Disorders, Schizophrenic" OR "Schizophrenic Disorder" OR "Dementia Praecox" OR "schizophrenia" ) ) AND ALL ( fmri OR "functional magnetic resonance imaging" OR mri OR "magnetic resonance imaging" OR "brain activity" OR "brain function" OR connectivity OR "neural activity" ) |
